# Dissociated Deficits between Explicit and Implicit Empathetic Pain Perception in Neurofibromatosis Type 1

**DOI:** 10.3390/brainsci11121591

**Published:** 2021-11-30

**Authors:** Hai Xue, Qiong Wu, Zhijun Yang, Bo Wang, Xingchao Wang, Pinan Liu

**Affiliations:** 1 Department of Neurosurgery, Beijing Tiantan Hospital, Capital Medical University, Beijing 100070, China; haixue@mail.ccmu.edu.cn (H.X.); zhijunyang@ccmu.edu.cn (Z.Y.); bowang@ccmu.edu.cn (B.W.); 2 China National Clinical Research Center for Neurological Diseases, Beijing 100070, China; 3 Beijing Key Lab of Learning and Cognition, School of Psychology, Capital Normal University, Beijing 100070, China; qwu@cnu.edu.cn; 4 Department of Neural Reconstruction, Beijing Neurosurgery Institute, Capital Medical University, Beijing 100070, China

**Keywords:** neurofibromatosis type 1, empathetic pain perception, cognitive impairment, social function deficits

## Abstract

Cognitive impairments and social-function deficits are severe complaints in neurofibromatosis type 1 (NF1) patients. Empathetic pain perception may be disrupted in NF1 patients because of high-level cognitive deficits. This study investigated the empathy profiles of adult patients with NF1, especially concerning whether explicit and implicit empathetic pain perception are abnormal in this population. We examined empathetic pain perception through a paradigm based on perceiving another person’s pain; in this task, patients were required to make judgments about the presence of pain or the laterality of the body part, as shown in a picture. Twenty NF1 patients without obvious social or communication difficulties completed the task, and the results were compared with results from the normal controls (NCs). Regarding explicit empathetic pain processing, i.e., judging the presence of “pain” or “no pain”, there were no significant differences between patients and controls in accuracy or reaction time. However, in implicit empathetic processing, i.e., judging the laterality of “pain” or “no-pain” pictures, NF1 patients had significantly lower accuracy (*p* = 0.038) and significantly higher reaction times (*p* = 0.004) than the NCs. These results were consistent with those of a previous study showing that high-level cognitive deficits were prominent in NF1 patients when performing challenging tasks. The mechanisms and related brain network activity underlying these deficits should receive attention in the future.

## 1. Introduction

Neurofibromatosis type 1 (NF1) is an autosomal-dominant genetic disorder with an incidence of approximately 1 in every 2700–3000 live births [1]. Typical manifestations of NF1 include pigmentary lesions (e.g., cafe-au-lait macules, skinfold freckling, and Lisch nodules in the iris), dermal neurofibromas, skeletal abnormalities (e.g., scoliosis, pseudarthrosis), and brain tumors (e.g., optic pathway gliomas) [1,2]. Social function deficits are common in patients with NF1, and these complaints can severely limit normal life functioning [3]. In addition, physiological and psychological comorbidities have been attracting attention for decades. According to previous studies, more than 30% of NF1 children may have comorbid attention-deficit/hyperactivity disorder (ADHD), and 13.2–29% of children with NF1 have comorbid autism spectrum disorder (ASD) [4,5,6].

Empathy is critical for effective social interactions, and any impairment in empathetic pain perception is a notable deficit in persons with diseases such as ASD and in those with focal brain lesions [7,8]. Empathetic pain perception is a form of integrative processing. Specifically, it involves correctly recognizing feelings of pain and then reacting appropriately [7,9]; this processing involves multiple brain areas and neural networks. Deficits in a wide range of cognitive processes as well as a high comorbidity with ASD may indicate that the empathetic pain perception of NF1 patients has been affected. However, the empathetic profiles of NF1 patients have not been developed.

In this study, we recruited 20 NF1 adult patients with no complaints of social interaction problems and 20 matched normal controls (NCs) to assess and compare empathetic pain perception using the “empathy for others’ pain” (EOP) task [7,8,9]. We hypothesized that adult NF1 patients may demonstrate deficits in empathetic pain processing, even though no clues have been apparent in daily conversation and communications. Identifying impairments in empathetic pain perception would help to clarify high-level brain dysfunction in NF1 patients without structural abnormalities. Furthermore, this study provides a new way to evaluate and explore certain brain networks. Based on these findings, new treatments and behavioral interventions could provide benefits that improve the quality of life of NF1 patients.

## 2. Methods

### 2.1. Patient and Normal Controls

Twenty-six NF1 adult patients were recruited through neurology and neurosurgical clinics involved in the study. All participants with NF1 had fulfilled the diagnostic criteria specified by the National Institute of Health Consensus Conference [10]. Four patients were excluded because they did not accomplish the task, and another two patients were excluded because severe depression was measured by the Beck Depression Inventory (with scores of 33 and 30 out of 39). Finally, 20 NF1 patients were included in the study. Meanwhile, 20 neurologically intact participants were recruited as the NC group. NCs were from local communities and matched with patients by age and education.

All participants were right-handed, without previous or current diagnosis of autism, ADHD, or related behavioral problems. The same experimental procedures were performed in all participants. Individual written consent was obtained from all participants, and the study was approved by the Institutional Review Board of the Beijing Tiantan Hospital, Capital Medical University (KY 2019-027-02).

Pre-task evaluations were previously described in [10]. All participants underwent the Mini-Mental State Examination (MMSE) and completed the short-form Beck Depression Inventory (BDI-SF). The MMSE is commonly used in clinical settings to screen for cognitive impairment. The highest possible score was 30 points. A score below 25 points indicates impaired cognitive function. Meanwhile, we used the 13-item BDI-SF to assess the general mood state of the participants [11]. The BDI-SF is appropriate for screening for depressive symptoms in medical patients and has been shown to have a good internal consistency. A higher score indicates a lower mood and more severe depression. MRI scans were performed in all patients to exclude the possibility of intracranial tumor. 

### 2.2. Empathy for Others’ Pain (EOP)

**Figure 1:**
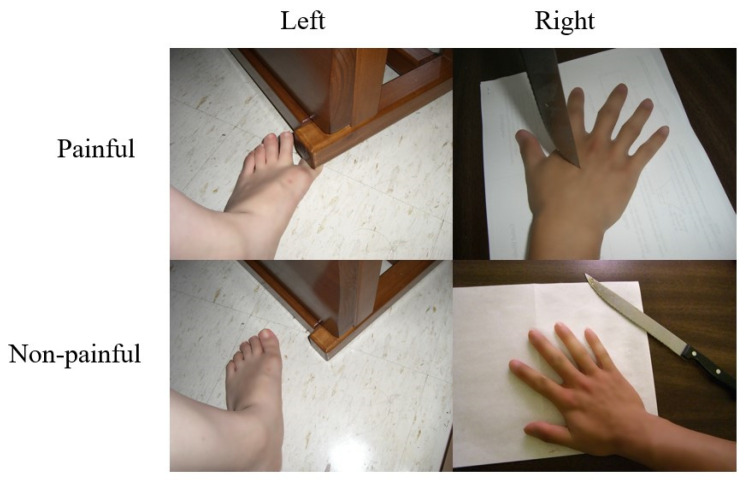
Sample stimuli from the experimental stimulus set. Patient and controls were instructed to choose between “no-pain” and “painful” for the “task–pain” sessions or between “left” and “right” for the “task–laterality” sessions by pressing the corresponding button within a time window of 4 s (2.5 s of stimulus presentation and 1.5 s of fixation).

Experimental stimuli and procedures were the same as those described in previous studies [7,8]. Briefly, participants viewed color photographs on a computer screen showing another person’s left or right hand or foot in painful or nonpainful situations. There were two types of tasks: in the task pain (TP) sessions, the subjects were instructed to judge whether the person in the photograph was suffering from pain; in the task laterality (TL) sessions, they were instructed to judge the laterality of the hand or foot (left or right) (see illustrations in Figure 1). Accuracy and reaction time (RT) were recorded.

### 2.3. Statistical Analysis

Accuracy and RT were analyzed for the four different conditions (TP-pain, TP-no pain, TL-pain, TL-no pain) in the EOP task. The participants’ sensitivity to pain and laterality was measured by the discrimination index d’, and decision bias was measured by the index β using signal detection theory (SDT) [12]. In the context of TP, d’ was the distance between the mean of the probability distribution for “pain” (target) and the mean of the probability distribution for “no pain” (noise), measured in units of standard deviations. β, which represented the position of the subject’s criterion, was the ratio of the height of the “pain” (signal) distribution to the “no-pain” (noise) distribution for the value of threshold. In the context of TL, d’ was the distance between the mean of the probability distribution for “left” (signal) and the mean of the probability distribution for “right” (noise), measured in units of standard deviations. β was the ratio of the height of the “left” (signal) distribution to the “right” (noise) distribution for the value of threshold. We calculated d’ and β for “pain” and “no-pain” conditions separately within the context of TL, and then the differences between the conditions were tested. Therefore, the d’ and β difference scores in TL represented the interference effects of pain on laterality judgments. The mean RT under TP ((RTTP-pain + RTTP-no pain)/2) and TL ((RTTL-pain + RTTL-no pain)/2) and the cost of pain in RT (additional time consumed in processing the pain information) under TP (RTTP-pain − RTTP-no pain) and TL (RTTL-pain − RTTL-no pain) were calculated. Paired two-tailed t-tests were performed for normally distributed data, and Mann–Whitney U-tests were performed for nonnormally distributed data. Only *p* < 0.05 was considered significant between the patient and control groups.

## 3. Results

### 3.1. Clinical Characteristics

The gender, age, education, BDI score, and MMSE characteristics are shown in Table 1.

All patients were free from cognitive impairment, as measured by the MMSE, and had slightly higher baseline BDI mood scores (indicating lower mood status) than the controls; the difference was close to significant (*p* = 0.095) (Table 1). Most of the patients suffered cutaneous disturbances without self-reported cognitive problems in daily life, and two of them complained of a slight memory decrease. The NF1 and NC groups were matched for age (*p* = 0.16) and gender (*p* = 0.74). The NF1 patients had an approximately two-year shorter education duration, but the difference was not significant.

### 3.2. Intact Explicit and Impaired Implicit Empathetic Pain Processing in NF1 Patients

For explicit empathetic pain processing under TP conditions, there were no significant differences between the patients and controls in d′ and β during pain judgment (results shown in Table 2). Neither the cost of pain RT nor the mean pain RT showed significant differences between the patient group and the NC group. Our results demonstrated no significant differences in the NF1 patients, as compared to the NCs, in the discrimination accuracy of others’ pain (indexed by d′ and β), and there were no significant changes in other behavioral indices during explicit empathetic processing.

**Figure 2:**
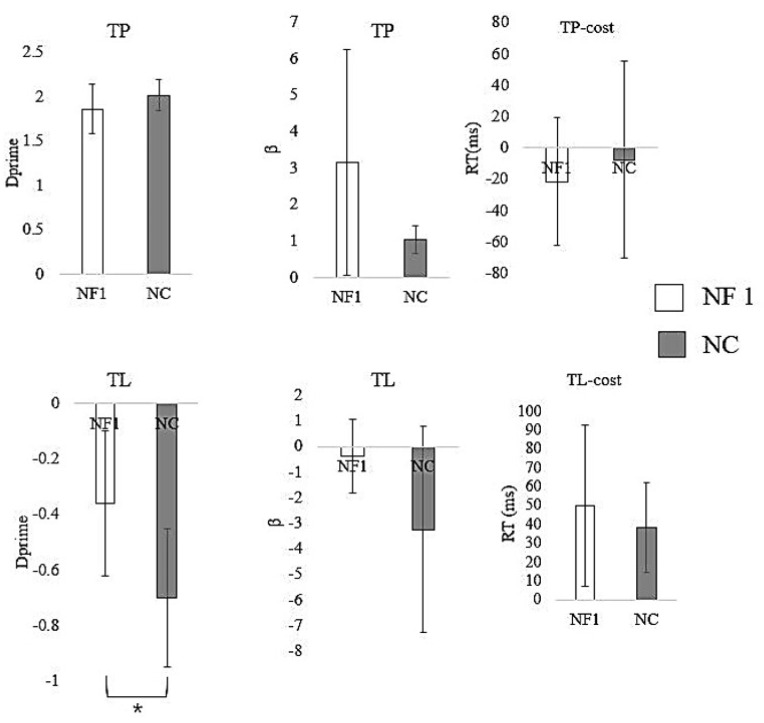
Behavioral performance on task–pain (TP) and task–laterality (TL). On TP, neither accuracy (d′) nor reaction time (RT) showed a significant difference between the NF1 patient group and the normal control (NC) group. In TL, NF1 patients showed significantly smaller d′ values than NCs (*p* = 0.038). Alteration in overall RT was close to significant between NF1 patients and NCs (*p* = 0.074). Error bars represent the 95% Confidence Interval (CI). * *p* < 0.05.

Implicit empathetic processing was examined by assessing the interference effect of empathetic pain on the laterality judgments (d’ TL-painful − d’ TL-non-painful). The NF1 patients showed significant differences in d’ scores (*p* = 0.038), as compared to the NCs, and a significant increase in the mean pain RT (*p* = 0.004). No significant difference was shown in β during pain judgment and cost of pain RT (details shown in Table 3 and Figure 2). These results indicated that implicit empathetic pain perception was disrupted in the NF1 patients, as compared to the NCs.

## 4. Discussion

In this study, we specifically focused on empathetic pain perception in adult NF1 patients. This research provided a subjective assessment that indicated abnormalities in empathetic pain perception and provided a possible reason for the social disability of NF1 patients.

### 4.1. Mechanisms Underlying Empathetic Pain Perception Deficits in NF1 Patients 

According to our results, the NF1 patients were comparable to normal controls in terms of explicit empathetic pain perception but showed deficits in implicit empathetic pain perception. In other words, they could appropriately feel another’s pain. However, when information on pain was integrated with other affective and/or cognitive information (as in our pain laterality task), the NF1 patients showed difficulties and deficits, suggesting dysfunction in the patients’ high-level processing networks. Few studies have focused on empathetic perception in NF1 patients. Experimental studies employing a similar strategy have been performed in NF1 patients. In a previous study, the Awareness of Social Inference Test (TASIT) was administered to adult NF1 patients and normal controls. In that study, the patients performed normally when recognizing direct sarcasm but were impaired in their ability to understand paradoxical sarcasm and sincerity. In addition, patients also had significant deficits in the recognition of facial expressions of emotion, especially anger [13]. NF1 patients have been reported to be relatively normal in terms of neutral face recognition but have shown significantly lower accuracy in identifying fear emotions in subsequent emotion-matching [14,15,16]. These results suggested that NF1 patients performed poorly on tasks that involved the feelings of other people during social interactions. This trend indicates that NF1 patients may face problems when stimuli are relatively complicated and indirect, suggesting that high-level cognitive impairment could exist in NF1 patients. Accordingly, we conducted this study to further evaluate high-level cognitive functions in these patients using empathetic pain-perception paradigms; this confirmed the existence of impairment, coordinating with our results that showed a dissociation between the faculties of implicit and explicit empathetic pain perception.

The mechanisms underlying this dissociation have been under debate, and we have highlighted three possible explanations. First, the differential responses under direct and indirect stimuli could be explained by the structural hypothesis that direct stimuli evoke the mirror neuron system, whereas indirect stimuli such as “facial emotion” are involved by an alternative process [17]. According to previous studies, deficits in explicit and implicit empathetic perception may be associated with the anterior insular cortex, as demonstrated by deficits in patients with anterior insular glioma [8,18,19]. However, other cortical regions such as the superior temporal gyrus and fusiform gyrus may be involved in higher social cognition and emotion recognition, respectively [20]. Certain networks could be affected in NF1 patients since multiple brain structural abnormalities other than a tumor could exist. Second, metabolic and signaling pathway changes in the brain may play important roles in behavioral abnormalities in NF1 patients [21]. For example, increased glutamate and GABA neurotransmission in the amygdala have been found in NF1 mice, whereas decreased levels of dopamine could result in attention deficits. Third, alexithymia, a condition characterized by a reduced ability to recognize, describe, and understand one’s own emotions, could co-occur with NF1. ASD patients with alexithymia exhibit obvious empathetic deficits [22]. Further neuroimaging studies could be performed in these patients to identify network abnormalities as well as any metabolic changes.

### 4.2. Cognitive and Social Problems in NF1 Patients

Cognitive and social difficulties in NF1 patients have drawn increasing attention in recent decades. When measured by IQ, general intelligence is slightly lower is these patients than in normative comparison groups, but it is usually only mildly affected. Similarly, the difference in general cognitive function measured by the MMSE between the NF1 patients and NCs was not significant in our study. The NF1 patients had a shorter education duration (10 years versus 12 years) than the NCs, but the difference was not significant. However, van der Vaart confirmed that a broad range of cognitive deficits and behavioral problems were detected in patients with NF1 across clinical trials [23]. Academic difficulties and school failure are the most commonly reported complications of NF1 in childhood, and these difficulties have been shown to stabilize in adulthood [3]. Previous studies have reported NF1 patients’ deficits in perception, attention, executive functioning, and language skills, which could result in social difficulties, anxiety, and even depression [24,25]. As in our study, NF1 patients showed higher BDI scores than the NCs.

Several studies have discussed the social difficulties of NF1 patients, mostly based on subjective scoring and questionnaires answered by others about the patients, providing multiple perspectives in various contexts. Scores from parents and teachers rated children with NF1 with lower scores on the social skills rating system (SSRS), indicating clinical social difficulties in NF1 children [26]. Ratings by family or friends using the social performance survey schedule revealed that NF1 adults showed less “eye contact when speaking” and less leadership, which were not reported by the patients themselves, suggesting reduced self-awareness and perception of their social difficulties. As a result, children with NF1 were rated by their peers as less well liked, were less often selected as a “best friend”, and had fewer reciprocated friendships [15]. Scoring scales related to such issues were not used in our study, but these evaluations remain important for the further detection and investigation of social problems in NF1 patients.

### 4.3. Future Considerations

Deficits in empathetic pain perception may result in social problems and communication difficulties, and more specific studies should be conducted to identify these problems in relation to NF1 patients. In our study, there was a limited assessment of ASD characteristics in our patients. In the future, such differentiation should be explicitly carried out. On the other hand, clinical trials aimed at improving cognitive deficits in NF1 patients have been reported in the literature. Methylphenidate has been shown to improve attention difficulties in trials [27,28]. Lovastatin, a cholesterol-lowering agent, improved learning deficits in NF1 mice but showed little improvement in patients [29,30]. Unfortunately, randomized controlled trials have indicated that simvastatin was similar to placebo in regard to all cognitive outcomes [31]. Drug therapy would be a promising direction for future treatment for NF1 patients.

Another research direction could be to identify the particular brain regions that contribute to emotion and social skills, such as empathy and anger. Functional MRIs should be attributive, and studies have been performed in several centers. Comparing patients and NCs could be an effective way to clarify the abnormalities and advance our understanding of brain function.

## 5. Conclusions

In summary, the current study suggested that implicit empathetic pain perception was disrupted in NF1 patients, as compared to the NCs, though patients exhibited normal explicit empathetic pain perception. This gives rise to the concern that social and cognitive deficits in NF1 patients only become apparent when complicated and challenging tasks are performed. The mechanisms and related brain network activity underlying these processes should be given special attention in the future.

## Figures and Tables

**Table 1:** General characteristics of NF1 patients and normal controls.

	NF1 Patients	Normal Controls	*p*-Value
Male–female	8:12	6:14	0.741
Age (X ± SD)	29.50 ± 8.35	33.55 ± 9.58	0.162
Education (years)	10.60	12.05	0.118
BDI	9.45	6.85	0.095 *
MMSE	28.85	27.75	0.124

* The Mann–Whitney U-test was performed.

**Table 2:** Group comparisons of explicit empathetic pain processing in NF1 patients and NCs.

Variables	NF1 Patients	Normal Controls	*p*-Value
TP d′	1.86	2.02	0.34
TP β	3.16	1.04	0.42 *
TP cost	−21.60	−7.50	0.53 *
TP mean	1083.55	1008.60	0.138

* The Mann-Whitney U-test was performed.

**Table 3:** Group comparisons on implicit empathetic pain processing in NF1 patients and NCs.

Variables	NF1 Patients	Normal Controls	*p*-Value
TP d′	−0.36	−0.70	0.038 *
TL β	−0.39	−3.23	0.588 *
TL cost	50.10	38.40	0.074 *
TL mean	1126.10	961.70	0.004

* The Mann–Whitney U-test was performed.

## Data Availability

The data presented in this study are available on request from the corresponding author. The data are not publicly available due to patients’ privacy and copyright issues.
